# Predicting the cost of malaria elimination in the Asia-Pacific

**DOI:** 10.12688/wellcomeopenres.15166.1

**Published:** 2019-04-24

**Authors:** Rima Shretta, Sheetal Silal, Lisa J. White, Richard J. Maude

**Affiliations:** 1 Global Health Group, University of California, San Francisco, San Francisco, CA 94158, USA; 2 Swiss Tropical and Public Health Institute, Basel 4002, Switzerland; 3 University of Basel, Basel 4001, Switzerland; 4 Modelling and Simulation Hub, Africa (MASHA), Department of Statistical Sciences, University of Cape Town, Rondebosch, Cape Town 7700, South Africa; 5 South African DST-NRF Centre of Excellence in Epidemiological Modelling and Analysis, Stellenbosch University, Cape Town, South Africa; 6 Centre for Tropical Medicine and Global Health, Nuffield Department of Medicine, University of Oxford, Oxford, Oxfordshire, UK; 7 Mahidol-Oxford Tropical Medicine Research Unit, Faculty of Tropical Medicine, Mahidol University, Bangkok, Bangkok 10400, Thailand; 8 Harvard TH Chan School of Public Health, Harvard University, Boston, MA, USA

**Keywords:** malaria, elimination, Asia-Pacific, economics, investment case

## Abstract

Over the past decade, the countries of the Asia-Pacific region have made significant progress towards the goal of malaria elimination by the year 2030. It is widely accepted that for the region to meet this goal, an intensification of efforts supported by sustained funding is required. However, robust estimates are needed for the optimal coverage and components of malaria elimination packages and the resources required to implement them. In this collection, a multispecies mathematical and economic modelling approach supported by the estimated burden of disease is used to make preliminary estimates for the cost of elimination and develop an evidence-based investment case for the region.

## Malaria elimination, transmission and costing in the Asia Pacific

The Asia Pacific region has made unprecedented gains against malaria with cases and deaths declining by more than 50% since 2000 ^1^. Strong political commitment and financial support from governments and donors has enabled the scale-up of effective interventions against the disease, facilitating the decline. However, these gains are fragile, and investments could be lost if malaria resurges. Although domestic financing for malaria has increased in many countries in the last decade, countries continue to rely on external financing to deliver high impact interventions particularly to vulnerable populations. At the same time, given the low levels of mortality in Asia, there is a risk of withdrawal of funding as the disease may no longer be perceived as a priority and there is mounting competition for the limited government resources from other pressing disease concerns. The growing threat of antimalarial drug resistance arising from the Greater Mekong Subregion and the risk of it spreading to other regions makes the case for malaria elimination an urgent priority. However, in order to achieve a malaria-free Asia Pacific – a goal endorsed by leaders at the highest levels though the Asia Pacific Leaders Malaria Alliance (APLMA) – financial resources will need to be sustained. Countries and partners need better estimates of the resources required to eliminate malaria in the long term, as well as evidence on the economic benefits of investing in its elimination in order to advocate for more resources.

## How much financing do we actually need to eliminate malaria?

There is limited understanding of the true volume of financing needed for malaria elimination due to a variety of reasons. Malaria programs in endemic countries are unable to capture the true burden of disease as data systems are often of poor quality and reporting is frequently incomplete. Furthermore, most information management systems do not capture the high volume of patients that access the often unregulated private sector and, in many countries, non-governmental organisations and therefore do not represent credible sources to estimate the financing needed for malaria services ^2^. For example, the burden of malaria in India has been estimated to be anywhere between 8 and over 100 times the reported burden ^3^, with the most recent estimate from the World Health Organization for 2017 being 8 to 15 times the reported burden, making a financial gap analysis challenging ^1, 3^.

Most country level estimates of need are obtained from National Strategic Plans (NSPs), which are often only available in countries eligible for financing from the Global Fund for HIV/TB and Malaria and are not always calibrated for elimination. Except for a few countries, these estimates of need do not incorporate interventions to improve efficiency or value for money; in many cases over-estimating the minimum costs required to deliver the plan. As NSPs are inadequate tools for cost estimation at the global level, models have been used to forecast need. While these have provided valuable projections of financing need in the absence of national level data, they have, to date, had limited applicability in Asia. Most of the models have been based on outputs of single species compartmental transmission models, almost exclusively focused on *Plasmodium falciparum* malaria transmission dynamics from sub-Saharan African countries. The ability of such a transmission model to simulate low or unstable levels of malaria transmission or the changing reservoir of parasites due to population movement and a diminishing population at risk is uncertain. They often assume universal coverage of all interventions and, as a result may overestimate costs. This may limit the models’ usefulness for Asia, which has higher proportions of *P. vivax*, a lower overall burden, and the impact of malaria interventions such as Long-Lasting Insecticide Treated Nets and Indoor Residual Spraying differ from what has been observed for *P. falciparum*.

APLMA was established at the 2013 East Asia Summit (EAS) to accelerate progress towards a reduction in malaria cases and deaths in the region. In 2014, EAS Heads of Government agreed to the goal of an Asia-Pacific region free of malaria by 2030 and a “Leaders’ Malaria Elimination Roadmap” was subsequently endorsed in 2015 ^4^.

As part of the evidence base for malaria elimination for the region, APLMA, with support from the Asian Development Bank, commissioned the Global Health Group at the University of California, San Francisco and Mahidol-Oxford Tropical Medicine Research Unit to estimate the burden of malaria in the Asia-Pacific region, predict the cost of elimination for a range of scenarios and develop an investment case for malaria elimination by 2030. This collection of papers presents the outputs from this work.

## Aims and scope

The objectives of this body of work were to:

- Estimate the true burden of malaria in the Asia Pacific region. The annual clinical burdens of disease for both *P. falciparum* and *P.* vivax malaria were estimated for the 22 countries from 2000 to 2015 ^5^.
- Develop a mathematical model to project the most epidemiological efficient rates of decline to elimination by 2030. As the Asia-Pacific region experiences both *P. falciparum* and *P. vivax* malaria, the mathematical model incorporated the dynamics and control measures for both species ^6^.
- Develop a software application that countries can use to visualize the effect of various scenarios of malaria control and elimination interventions to predict the optimal path to elimination in the region ^7^.
- Estimate the cost to achieve malaria elimination in the Asia Pacific region by 2030 based on the above and to determine the economic impact of interventions against the transmission of *P. falciparum* and *P. vivax* malaria; generate an investment case for malaria by estimating the economic benefits of malaria elimination and prevention of reintroduction and; identify the funding gaps and explore the potential opportunities for generating financial resources for achieving malaria elimination goals ^8^.

## Data availability

No data is associated with this article.
